# Mesenchymal stromal cells from a progressive pseudorheumatoid dysplasia patient show altered osteogenic differentiation

**DOI:** 10.1186/s40001-022-00683-2

**Published:** 2022-04-25

**Authors:** Lia Pulsatelli, Cristina Manferdini, Elena Gabusi, Erminia Mariani, Francesco Ursini, Jacopo Ciaffi, Riccardo Meliconi, Gina Lisignoli

**Affiliations:** 1 Laboratorio di Immunoreumatologia e Rigenerazione Tissutale, IRCCS Istituto Ortopedico Rizzoli, via di Barbiano 1/10, 40136 Bologna, Italy; 2 Dipartimento di Scienze Mediche e Chirurgiche, Alma Mater Studiorum, Università Di Bologna, Bologna, Italy; 3 Medicina e Reumatologia, IRCCS Istituto Ortopedico Rizzoli, Via Pupilli 1, 40136 Bologna, Italy; 4 Dipartimento di Scienze Biomediche e Neuromotorie, Alma Mater Studiorum, Università di Bologna, Bologna, Italy

**Keywords:** Progressive pseudorheumatoid dysplasia, Mesenchymal stromal cells, Osteoblasts, Osteogenic markers

## Abstract

**Background:**

Progressive pseudorheumatoid dysplasia (PPRD) is a rare autosomal recessive non-inflammatory skeletal disease with childhood onset and is characterized by a progressive chondropathy in multiple joints, and skeletal abnormalities. To date, the etiopathological relationship between biological modification occurring in PPRD and genetic mutation remains an open issue, partially due to the limited availability of biological samples obtained from PPRD patients for experimental studies.

**Case presentation:**

We describe the clinical features of a PPRD patient and experimental results obtained from the biological characterization of PPRD mesenchymal stromal cells (MSCs) and osteoblasts (OBs) compared to normal cell populations. Phenotypic profile modifications were found in PPRD compared to normal subjects, essentially ascribed to decreased expression of CD146, osteocalcin (OC) and bone sialoprotein in PPRD MSCs and enhanced CD146, OC and collagen type I expression in PPRD OBs. Gene expression of Dickkopf-1, a master inhibitor of WNT signaling, was remarkably increased in PPRD MSCs compared to normal expression range, whereas PPRD OBs essentially exhibited higher OC gene expression levels. PPRD MSCs failed to efficiently differentiate into mature OBs, so showing a greatly impaired osteogenic potential.

**Conclusions:**

Since all regenerative processes require stem cell reservoirs, compromised functionality of MSCs may lead to an imbalance in bone homeostasis, suggesting a potential role of MSCs in the pathological mechanisms of PPRD caused by WNT1-inducible signaling pathway protein-3 (WISP3) mutations. In consideration of the lack of compounds with proven efficacy in such a rare disease, these data might contribute to better identify new specific and effective therapeutic approaches.

## Background

Progressive pseudorheumatoid dysplasia (PPRD) is a rare autosomal recessive non-inflammatory skeletal disease with childhood onset, which is characterized by a progressive chondropathy affecting multiple joints, and skeletal abnormalities.

Typical radiological signs include enlargement of the epimetaphyseal parts of large joints, metacarpal heads, and phalanges. The spine shows a generalized platyspondyly with irregular delineation of vertebral endplates [1] and magnetic resonance imaging (MRI) examination indicates enhanced bone metabolism [1, 2]. Imaging and histological examination reveal changes that resemble common, end-stage osteoarthritis [2–4]. Frequently, due to these joint alterations, PPRD patients need joint replacement surgery by the third decade of life [1].

Progressive pseudorheumatoid dysplasia is recognized to be associated with mutations in the WNT1-inducible signaling pathway protein-3 (WISP-3) gene [4–6].

The WISP3 gene belongs to the CCN family of proteins that are involved in the regulation of cell growth, differentiation, development, communication, and response in multiple cell lines [7]. In particular, WISP3 has been shown to mediate up-regulation of collagen type II and aggrecan in human chondrocyte cell lines. Its role in cartilage integrity and homeostasis has been demonstrated by studies that reported cellular and molecular modification in chondrocytes obtained from a PPRD patient harboring the WISP3 mutation [6] and in human chondrocyte cell lines transfected with WISP3 mutants [8].

Furthermore, WISP3 is a chemoattractive ligand for human multipotent mesenchymal stromal cells (MSCs) [9] and it has been recognized as a modulator of bone morphogenetic proteins (BMPs), WNT and Notch1 signaling [4, 10]. All three signaling pathways are directly involved in the regulation of the entire osteoblastic lineage from MSC commitment up to terminal osteoblast (OB) differentiation [11], by tuning osteoprogenitor pool plasticity and contributing to bone formation, turnover and repair.

Therefore, we focused on the biological and molecular changes in PPRD MSCs and OBs to obtain new insights into these aspects, which may provide a valuable basis for the clarification of the etiopathological mechanisms of PPRD caused by WISP3 mutations, an issue which currently remains open.

On these bases, we isolated MSCs and OBs from bone samples from a PPRD patient for phenotypic, molecular, and functional analyses, focusing on:

- specific positive/negative membrane-bound molecules that characterized osteoprogenitor-committed MSC and OB phenotypes, such as CD105, CD146, CD34;
- a panel of biomarkers associated with osteoblastic lineage, including alkaline phosphatase (AP), extracellular matrix collagenous proteins (collagen type I, collagen XV) and extracellular matrix non collagenous proteins (osteocalcin—OC, bone sialoprotein-BSP);
- Dickkopf-1 (DKK1), a master inhibitor of WNT signaling.

We then investigated the extent of the osteogenic potential of MSCs, evaluating their ability to differentiate into mature osteoblasts capable of forming mineralized matrix.

Here we describe the clinical features of a PPRD patient and the experimental results obtained from the biological characterization of PPRD MSCs and OBs.

## Case presentation

### Clinical history

We report the case of a female patient with a molecular genetic diagnosis of PPRD made in 1999, who was first seen in our unit in 2006, at the age of 41.

The first symptoms appeared in childhood, with joint pain and swelling accompanied by fever and fatigue. A diagnosis of Juvenile Idiopathic Arthritis was proposed and treatment with IV gold was commenced when she was 8 years old, achieving symptomatic relief after few weeks of therapy. After 12 months, gold therapy was discontinued due to mild side effects and sustained clinical remission. Over the following years, the patient complained of diffuse joint pain with primary involvement of the hips and knees, and poor pain relief from non-steroidal anti-inflammatory drugs (NSAIDs).

**Fig. 1:**
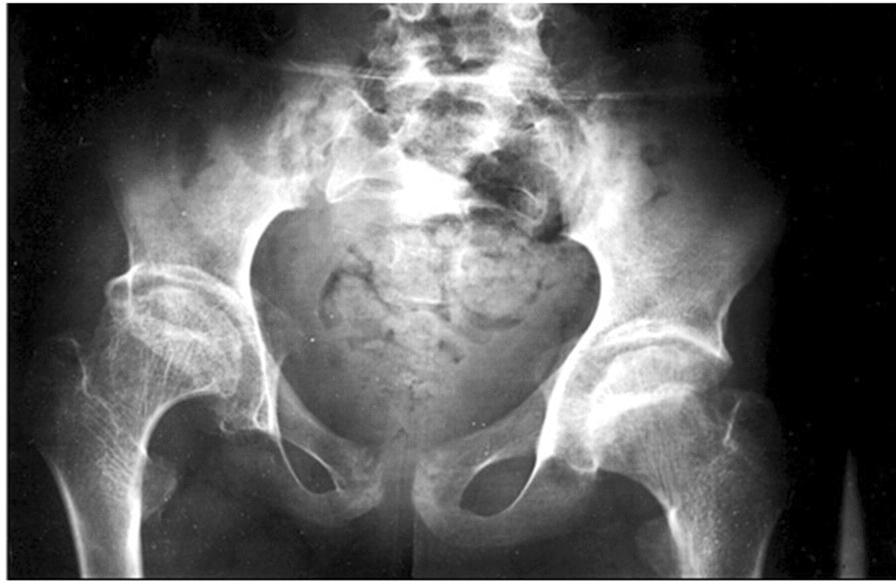
X-ray of the pelvis of the PPRD patient that reveals severe hip osteoarthritis

We performed X-rays of the patient's hands, shoulders, elbows and spine, which revealed diffuse modifications of the articular cartilage and bone epiphyses, as we previously reported [12, 13] Wide epiphyses and enlarged metaphyses were evident in the metacarpophalangeal and interphalangeal joints [12, 13], while severe hip osteoarthritis (Fig. 1) and characteristic platyspondyly of thoraco-lumbar spine were also noted [12]**.**

A deterioration in hip mobility and worsening of pain due to early secondary osteoarthritis led to right and left total hip and knee arthroplasties (1994, 1997, 2010, and 2012, respectively).

### Biological investigations: phenotypic, molecular, and functional evaluations

Mesenchymal stromal cells and OBs were isolated from the tibia plateau obtained from the PPRD patient and six subjects undergoing total knee replacement for traumatic events who were considered as normal controls (three males, three females; mean age ± standard deviation: 45 ± 7 years). Written informed consent was obtained from the patients/participants.

The phenotypic profile of both MSCs and OBs was characterized by flow cytometry analyzing the following markers: CD34, CD105, CD146, collagen type 1, OC, BSP and AP and data expressed as percentage of positive cells are reported in Table 1.

**Table 1:** Phenotypic characterization of MSCs and OBs from a PPRD patient and normal subjects

Markers	PPRD MSC %	Normal MSC % median (range)	PPRD osteoblasts %	Normal osteoblasts % median (range)
CD34	0	1 (1–2)	2	2 (1–2)
CD105	100	98 (94–100)	100	98 (45–100)
CD146	**4**	30 (15–37)	**27**	2 (1–9)
Collagen type I	88	79 (35–100)	**96**	36 (4–94)
OC	**60**	87 (75–95)	**100**	53 (6–81)
BSP	**17**	40 (29–83)	25	24 (3–90)
AP	9	5 (1–29)	14	22 (2–38)

PPRD markers out of normal range are indicated in bold

*OC* osteocalcin, *BSP* bone sialoprotein, *AP* alkaline phosphatase

In the PPRD patient and the normal subjects, MSCs and OBs were negative for CD34, a typical hematopoietic marker, which should be absent in MSCs and OBs isolated in vitro, whereas CD105 (a phenotypic key marker shared by MSCs and OBs), was similarly expressed by the PPRD and normal cell populations.

CD146 is recognized as a specific marker expressed by MSCs, but not by mature osteogenic cell strains. CD146-positive MSCs were sharply lower in PPRD MSCs compared to the normal range, conversely CD146-positive cells were considerably increased in PPRD OBs compared to the normal OB expression range. This specular modification resulted in an opposite expression pattern in PPRD MSCs and OBs compared to normal controls. Indeed, in normal subjects, CD146 appeared to be clearly more highly expressed in MSCs than in OBs, whereas in PPRD, CD146 expression was higher in OBs than in MSCs.

Furthermore, compared to the expression range estimated in normal subjects, PPRD MSCs were characterized by a decrease in OC and BSP-positive cells, whereas the percentages of collagen type 1 and AP positive cells were within normal range.

Progressive pseudorheumatoid dysplasia OBs exhibited an increased percentage of OC and collagen type I positive cells, whereas the percentage of BSP and AP positive cells were within the normal range.

In addition to investigations at protein level, collagen type I, OC, BSP, AP, were evaluated at gene expression level, together with collagen type XV and DKK-1, by real-time quantitative reverse transcriptase polymerase chain reaction (RT-PCR). For each target gene, mRNA levels were calculated, normalized to GAPDH housekeeping gene and expressed as a percentage of the reference gene.

**Fig. 2:**
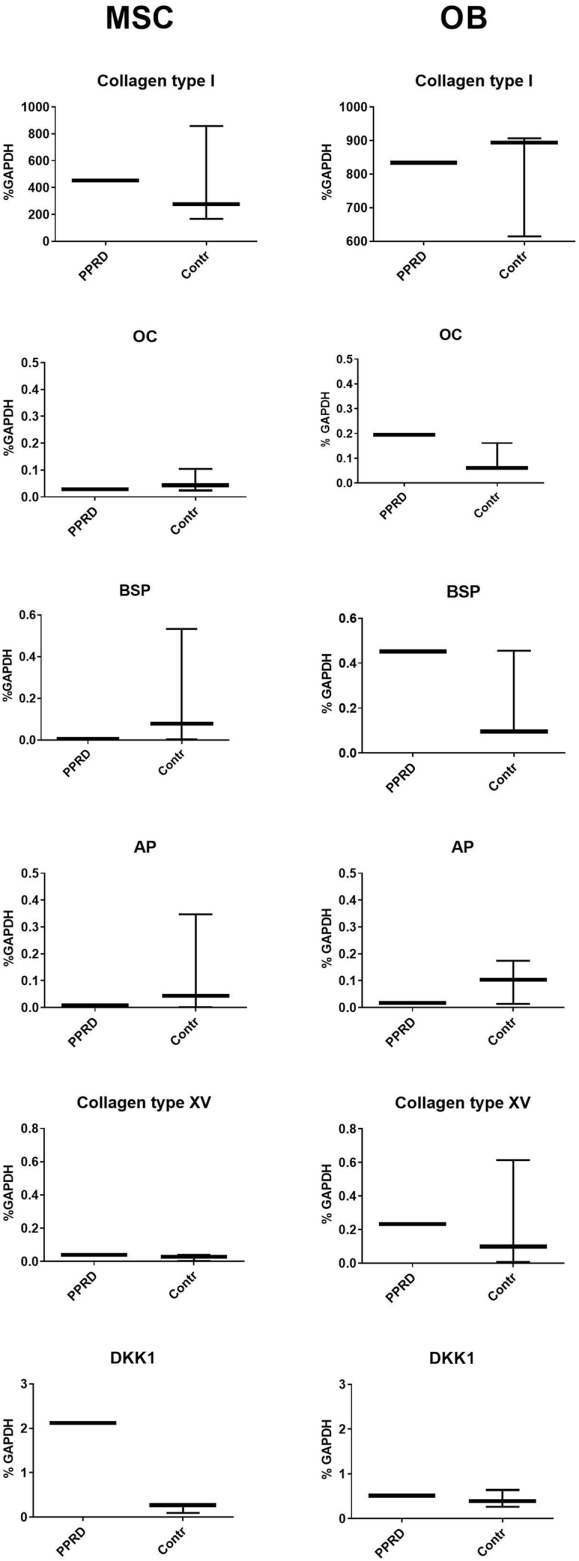
Gene expression analysis of collagen type I, osteocalcin (OC), bone sialoprotein (BSP) alkaline phosphatase (AP), collagen XV, Dickkopf-1 (DKK1). Data are expressed as percentage of the GAPDH reference gene. Boxes indicate the 25th and 75th percentiles, whiskers indicate the minimum to maximum values, and bars indicate the median

As reported in Fig. 2, we observed that in PPRD MSCs only DKK-1 gene expression appeared to be considerably increased compared to the expression range estimated in normal MSCs.

On the other hand, in PPRD OBs, only the OC gene expression level was above the expression range observed in normal subjects, confirming findings detected at protein level.

Osteocalcin expression in MSCs, along with collagen type I, BSP, AP and collagen type XV gene expression, both in MSCs and OBs, did not show relevant deviations compared normal ranges. Furthermore, Collagen type I, OC, BSP and AP gene expression showed the same trend and the same reciprocal fluctuation detected at protein level both in MSCs and OBs.

Finally, we induced osteogenic differentiation of MSCs by maintaining them in culture with osteogenic medium for up to 21 days. The extent of the osteogenic potential of MSCs for forming mineralized matrix was evaluated and visualized by Alizarin red staining.

**Fig. 3:**
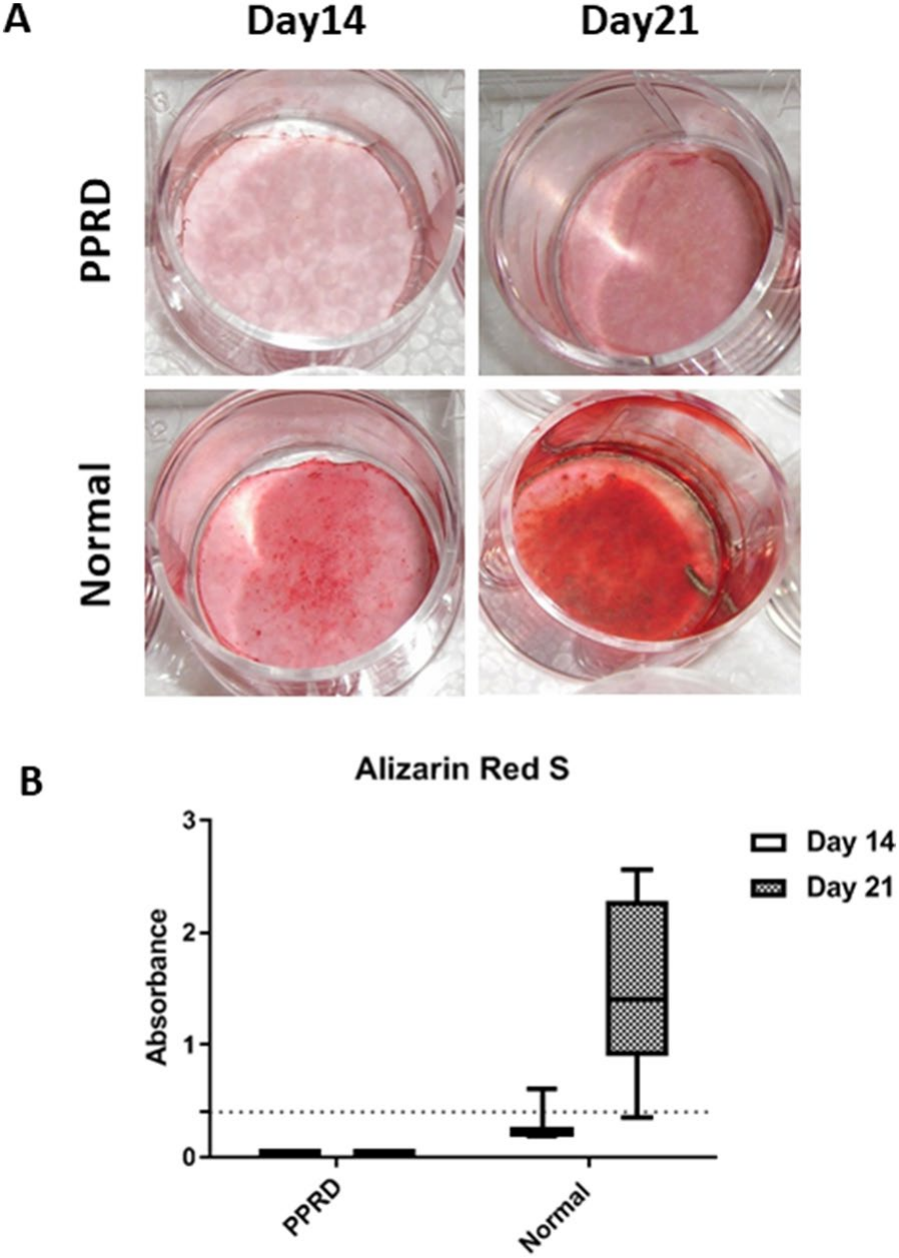
Deposition of mineralized matrix by Alizarin red staining. **A** Representative images of negative and positive staining in osteogenic differentiated mesenchymal stromal cells from PPRD patient and normal subjects. **B** Spectrophotometric quantification Boxes indicate the 25th and 75th percentiles, whiskers indicate the minimum to maximum values, and bars indicate the median. Dotted line indicates detection limit

Progressive pseudorheumatoid dysplasia MSC cultures were found to be negative for Alizarin red staining, failing to efficiently differentiate in mature OBs, thus showing a greatly impaired osteogenic potential (Fig. 3).

## Discussion

The etiopathological relationship between the biological modification occurring in PPRD and the genetic mutation remains, to date, an open issue, also due to the very limited availability of biological samples obtained from PPRD patients for experimental studies.

We focused on bone niche, exploring the phenotypic, molecular, and functional features of MSC osteolineage progenitors and mature OBs obtained from a PPRD patient, in order to evaluate any potential biological modification compared to normal cell populations.

Bone and skeleton integrity is qualitatively and quantitatively maintained by a constant remodeling of its architecture and composition. Bone homeostasis requires a perfect balance between the resorption phase and the replacement of new bone by OBs. The imbalance in these two processes results in skeletal abnormalities [14].

Osteoblasts differentiate from MSCs through a process organized in tightly tuned steps that begins with MSC recruitment, followed by their ability to respond to osteogenic stimuli and finally to OB commitment, maturation, and extracellular matrix mineralization [14].

Mesenchymal stromal cells are defined as self-renewable, multipotent progenitor cells that are characterized by the presence of membrane-bound proteins including, CD105, CD146, CD73, and CD90 markers, and by the absence of hematopoietic antigens such as CD34, CD45, CD14 [15, 16]. Unfortunately, the identification of specific profile markers that univocally identify MSCs remains controversial, since it has been recognized that MSC key markers may also be expressed by OBs, pointing out the high plasticity of these cells [16], as also highlighted by our results.

Our finding showed that CD146-positive MSCs were sharply lower in PPRD than in normal subjects, conversely, CD146-positive cells were considerably increased in PPRD OBs compared to normal OBs. Changes in CD146 expression have been recognized to be associated with pivotal MSC functional modifications. In particular, decreased CD146 expression appeared to be associated with an impaired migration potential [17] and the compromised ability of aged MSCs to differentiate toward osteogenic lineage [18]. This latter finding is in line with our results, which demonstrated a greatly impaired osteogenic potential of PPRD MSCs.

Furthermore, since CD146 expression decreased after osteogenic induction [15], the increased CD146-positive percentage of PPRD OBs might underline an alteration in their differentiation condition, with a phenotypic transition toward an earlier lineage stage.

This hypothesis appears to be supported, to the best of our knowledge, by the only study previously performed on OBs obtained from a PPRD patient [19], which underlined an increased cell proliferation rate, a typical feature of immature status, since proliferative capacity decreases according to increasing cell differentiation [14]. In addition, an altered differentiation has also been reported in PPRD chondrocytes. Indeed, Zhou et al. [6] showed that these cells proliferated significantly faster than normal cells, thus indicating an immature and hyper-proliferative state of PPRD chondrocytes.

In addition, our data showed bone marker profile modifications in PPRD MSCs and OBs, essentially ascribed, at protein level, to decreased expression of OC, BSP in PPRD MSCs and enhanced OC and collagen type I expression in PPRD OBs.

These findings corroborate the hypothesis of an altered differentiation process of OB lineage cells in PPRD. In fact, it has been shown that the differential tightly regulated expression of bone marker genes results in a peculiar protein profile associated with the specific sequential phases of the bone differentiation process, characterized by osteoprogenitor proliferation to differentiation, maturation and mineralization [20].

Furthermore, since WNT pathway components are essential for OB lineage development and maturation [21] and WISP3 has been recognized as WNT signaling modulator [4], we investigated gene expression of DDK-1, one of the major inhibitors of WNT signaling. Interestingly, WISP3 has been shown to modulate WNT signaling by binding to WNT co-receptor low-density lipoprotein receptor-related protein 6 (LRP6) [4], which is the same co-receptor engaged by DKK-1 to block further interaction with WNT ligands [21].

DKK-1 gene expression was shown to be remarkably increased in PPRD MSCs compared to the estimated normal expression range. Interestingly, increased DKK-1 mediates the impaired osteogenic capacity of adipose tissue-derived MSCs in steroid-induced osteonecrosis [22]. This evidence is consistent with our results, since PPRD MSCs, which were shown to express elevated DKK-1 at the molecular level, failed to efficiently differentiate into mature OBs.

## Conclusions

Our overall results, which demonstrate that phenotypic and molecular modifications occur in PPRD MSCs and OBs, might suggest a dysfunctional differentiation process affecting these cells, highlighted by the functional changes of MSCs, which show a greatly impaired osteogenic potential.

Considering that WISP3 can function as a chemotactic signal for human MSCs [9], WISP3 mutation may affect MSC migration, a key early step in bone formation. Indeed, MSCs need to migrate to the bone surface before initiating differentiation into osteogenic cells [21], consequently the altered migration ability of MSCs may lead to a homeostasic imbalance of bone.

This evidence, along with the results obtained from our study, which explored the biological characteristics of PPRD MSCs, highlights that, since all regenerative processes require stem cell reservoirs, compromised functionality of MSCs may lead to an imbalance in bone homeostasis, thus suggesting a potential role of MSCs in the pathological mechanisms of PPRD caused by WISP3 mutations.

The association among WISP mutation and functional/phenotypical alteration in PPRD remains to be explored and needs further investigations. Nevertheless, our novel results may be considered a valuable basis for complementary future investigations aimed to better understand the pathogenesis of PPRD and to identify new specific and effective therapeutic approaches, considering the lack of compounds with proven efficacy in such a rare disease.

## Data Availability

The datasets used in this study are available from the corresponding author on reasonable request.

## Abbreviations

PPRD: Progressive pseudorheumatoid dysplasia
MRI: Magnetic resonance imaging.
WISP-3: WNT1-inducible signaling pathway protein-3
MSCs: Mesenchymal stromal cells
BMPs: Bone morphogenetic proteins
OB: Osteoblast
AP: Alkaline phosphatase
OC: Osteocalcin
BSP: Bone sialoprotein
DKK1: Dickkopf-1
RT-PCR: Real-time quantitative reverse transcriptase polymerase chain reaction analysis
